# Effects of First Wave of COVID-19 on Colon Cancer Multi-disciplinary Team Performance: A Two-Year Analysis

**DOI:** 10.31661/gmj.v13i.3305

**Published:** 2024-02-26

**Authors:** Kulkarni Gaurav Vidyadhar, Kumaran Narenkumar, Kelkar Ashish Prabhakar

**Affiliations:** ^1^ Department of General and Colorectal Surgery, Kettering General Hospital, Kettering, East Midlands, United Kingdom

**Keywords:** Bowel Cancer, Colon Cancer, COVID-19, Survival Rate

## Abstract

Background: We investigated the effects of COVID-19-related delay on two-year outcomes of colon cancer treatment during the first wave of the pandemic. Materials and Methods: Ninety-two patients were referred for bowel cancer at our National Health Service (NHS) trust between March and July 2020, and 41 patients were treated for colon cancer and followed up (a two-year) through a multidisciplinary team (MDT). Treatment delays and overall survival (OS) were also assessed. Results: Treatment delays were observed in 48% of patients. The average delay was 31 days beyond the 62-day mark (P0.001). Logistic and binary logistic regression models showed that a comorbid diagnosis of respiratory disease had a significant effect on delays in management and two-year outcomes (P=0.04), but without the likelihood of upstaging or a poorer outcome (P=0.942). The overall survival rate was 81.5%. Eight percent of bowel cancer surgeries could have been avoided if endoscopic visualization and biopsy were available, and 8% more surgeries could have been performed laparoscopically without fear of surrounding aerosols. Conclusion: The findings showed that oncologic care provided minimal disruption to trust during the COVID-19 pandemic owing to a quick association between the NHS site and a green non-NHS site, resulting in acceptable two-year outcomes for colon cancer patients.

## Introduction

Cancer of large bowel (CRC) are in the top five most common malignancies in the United Kingdom (UK). There are about 16,800 bowel cancer deaths in the UK every year, which translated to 46 deaths every day between 2017 and 2019 [1]. Prognosis appears to be associated with the stage at time of diagnosis, making early suspicion referrals—via two-week wait (2WW) pathway, the Multi-Disciplinary Team (MDT) or diagnosis via the Bowel Cancer Screening Programme (BCSP)—being the best-case scenarios for improved survival [2].

Surgery to remove segment of the bowel is the central component of CRC management, followed by chemotherapy as applicable [3][4]. The world was affected by COVID-19 in 2020. It was labelled a global pandemic by the World Health Organization (WHO) in March 2020 [5][6]. The pressure of this led to the changes in thousands of elective appointments across the UK, even for urgent issues like cancer. An estimated 650,000 patients undergoing cancer care were affected due to the pandemic from staff redeployment, sickness, or isolation among essential staff, or to the uncertainty surrounding aerosol generating procedures (AGPs), leading to novel approaches in care [7][8]. Few studies have correlated deviations in the cancer management pathway with long-term outcomes. Therefore, this study aimed to present data from patients with colon cancer managed by our trust during the first wave of the pandemic and the deviations from the 2WW pathway and their outcomes two years after the completion of treatment, which is considered a significant point of follow-up in CRC care.

## Materials and Methods

**Table:** Table1. Demographic and Clinical Characteristics of Included Patient

	Patient Demographic and characteristics			
Sr. no	Parameter		No. of patients	p value
1	Gender	Male	19	0.213
		Female	22	0.132
		Grade 1	13	0.333
2	ASA grading	Grade 2	19	0.19
		Grade 3	9	0.091
		PS 0	16	0.131
3	Performance status	PS 1	17	0.235
		PS 2	8	0.1
		Hypertension	14	0.133
		IHD	10	0.65
		COPD/Asthma	7	0.040
4	Comorbidities	Diabetes	6	0.145
		Smoking	4	0.222
		Heart failure	3	0.325
		Obesity (BMI>35)	3	0.645

**ASA**=American Society of Anaesthesiology, **IHD**=ischemic heart disease, **COPD**=chronic obstructive pulmonary disease, **BMI=**body mass index

Study Population

This study was conductd on patients referred for bowel cancer at our National Health Service (NHS) trust between March 1, 2020, and July 31, 2020. A total of 41 patients were treated for colon cancer and followed up (a two-year period) by a multidisciplinary team (MDT). The regional CRC MDT at our trust was suddenly and significantly affected by March 2020. Being a moderate-sized NHS foundation trust and only one of two district general hospitals serving an entire county, we had to make some urgent decisions in line with the guidelines given by the Royal College of Surgeons (RCS) and the Association of Coloproctology of Great Britain and Ireland (ACPGBI) [9].

Inclusion and Exclusion Criteria

The participants in our study were adult patients referred to the CRC MDT at our trust and discussed it from the standpoint of curative intent, watchful waiting, or deferring plans after the pandemic. Patients who received palliative care or best supportive treatment were excluded.

We included patients who would otherwise be considered acceptable risks, but who were considered to be at high risk for COVID-19 exposure. However, we excluded patients who were considered unfit for surgery or managed outside the 2WW referral system. We excluded rectal cancer referrals because our center was part of a national audit during COVID-19 in the IMPACT-ReCaP study [10].

Study Design

Our study was a single-center chart review of a prospectively maintained database, using registry data permitted by the institutional audit committee.

Owing to a smaller referral base, specific geographical locations, and ties with a local private hospital network, we were able to rapidly perform significant alterations to our usual cancer pathway during the first wave of COVID-19 to keep cancer services fully functional in a green corridor. Our operative case backlog was at a minimum as the country emerged from the first wave of COVID-19 and entered the immunization era. Treatment delay was defined according to the NHS guidelines as one of two parameters: delay outside the standard 2WW pathway and delays greater than 62 days in total for the start of treatment. We reported these data in line with the 2021 STROCSS criteria.

Data Collection

Demographic variables obtained from the clinical and electronic records at the primary NHS site and satellite site (private non-NHS hospital) were included in logistic regression analyses (Table-1). Clinical variables included tumor size, node, metastasis (TNM), tumor location, and dates indicating delays. The surgery was reported as either an open or minimal access surgery. The histopathological results were reported according to the TNM/AJCC system.

Statistical Analysis

The categorical variables were expressed as percentages. Logistic regression analysis was used to assess a comorbid diagnosis of chronic obstructive pulmonary disease (COPD)/asthma and its effect on COVID-19-related delays in management and on two-year outcomes. All analyses were performed using the SPSS statistical package version 23 (San Diego, California, USA). Statistical significance was assumed at P<0.05.

## Results

**Figure-1:**
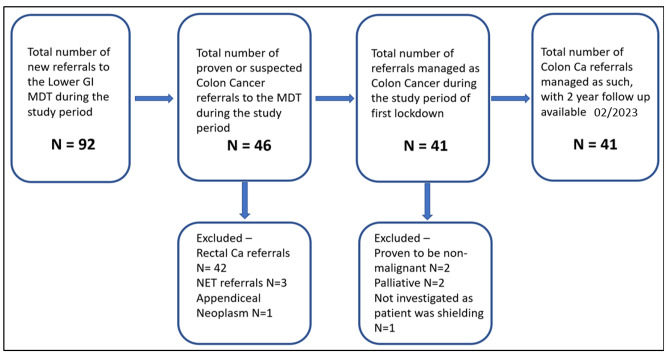
Figure 1. Flow Diagram for Inclusion, Exclusion and Follow Up of Study Participants (NET=Neuro Endocrine Tumor).

**Figure-2:**
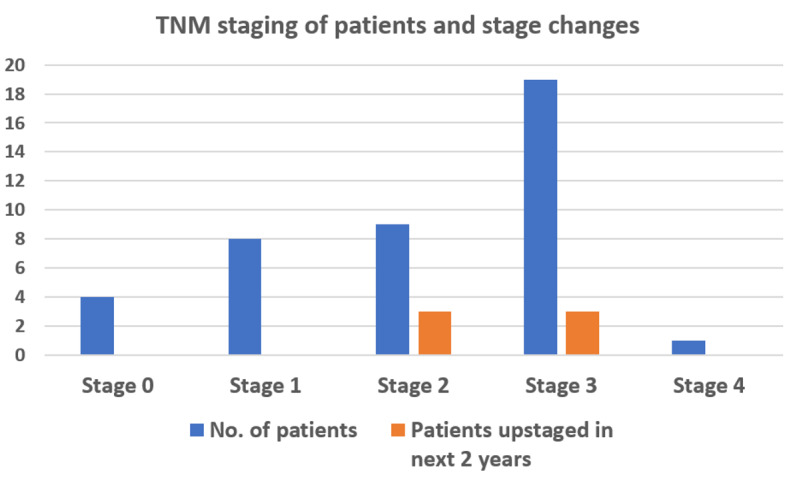
Figure 2. Tumor, node, metastasis (TNM) staging of patients and stage change data over the two years following completion of treatment.

**Table:** Table2. Binary logistic Regression Model for COVID-related Variables Affecting Delays in Management and Outcomes.

			Binary Logistic Regression		
Sr. no	Independent variables	Dependent variable	Individual p value of the independent variables	p value for the regression model	Interpretation
	Covid related delay in investigations		0.015		
1	Previous Covid-19 diagnosis prior to MDT referral	Covid-19 related delay in treatment	0.947	0.584	The regression model does not have a significant p value and hence, the said independent variables do not significantly affect the COVID-19 related delay in treatment
	2WW breach due to Covid-19 prior to decision		0.556		
	Was surgery at main usual NHS hospital		0.313		
2	Hospital course complicated by Covid-19	Patient whether alive at 18 months	0.225	0.539	The regression model does not have a significant p value and hence, the said independent variables do not significantly affect the patient outcome in terms of death
	Length of stay in hospital		0.257		
	Previous COVID-19 Diagnosis prior to MDT referral		0.999		
3	2WW breach due to Covid-19 prior to decision	Patient whether alive at 18 months	0.942	0.875	The regression model does not have a significant p value and hence, the said independent variables do not significantly affect the patient outcome in terms of death
	Would treatment plan be different in non-Covid times		0.652		

Ninety-two patients were referred to our lower gastrointestinal MDT during this period, of whom 46 were referred to as having proven or suspected colon cancer. Of these, 41 (19 male and 22 female) were managed (Figure-1). The mean age of the participants was 70 years (median 71.2 years). Thirty-four patients (73.9%) were above the age of 60 years and 22 were either current or past smokers. Twelve patients underwent surgery at the usual NHS location, while 29 underwent surgery at a non-NHS private location. Logistic regression showed that a comorbid diagnosis of COPD/asthma had a statistically significant effect on COVID-19-related delays in management and on two-year outcomes (P=0.04). The effects of none of the other demographic variables were significant (Table-1). Two patients underwent Hartmann’s procedure instead of sigmoidectomy and anastomosis because their comorbidities put them at high risk of COVID-19 in case they needed ITU care perioperatively, either during the first surgery or in case of a leak. One of these patients subsequently underwent uneventful laparoscopic reversal of the stoma. The patient did not require any further surgery. Five patients had their operation performed open instead of laparoscopically for concerns surrounding aerosolized spread of SARS-CoV-2 during the initial days of the pandemic due to the unavailability of air seals.

Eleven patients were offered adjuvant chemotherapy, but three refused in view of COVID-19 risks from immunosuppression and repeated hospital visits. Five patients completed adjuvant chemotherapy, of which four were alive at the end of two years despite two having upstaging in their second year and therapy for it. One of the three patients who refused adjuvant therapy died at the end of two years but the numbers were too small to calculate significance. Two patients received palliative chemotherapy and one patient accepted the plan. However, both died at the end of the two years. Three patients underwent right hemicolectomy for suspicious imaging findings suggestive of malignancy. One patient underwent sigmoidectomy for a three-centimetre suspicious sessile polyp found on CT colonography performed just prior to lockdown. All four had a final histology, yielding benign findings, such as two colonic lipomas and two large polyps with low-grade dysplasia. These surgeries could have been potentially avoided if colonoscopy had been performed with direct visualization and histologic diagnosis with endoscopic mucosal resection or complex polypectomy without fear of surrounding AGPs.

Two patients underwent Hartmann’s procedure instead of sigmoidectomy and anastomosis because their comorbidities put them at high risk of COVID-19 in case they needed ITU care perioperatively either during the first surgery or in case of a leak. One of these patients subsequently underwent uneventful laparoscopic reversal of the stoma. The patient did not want further surgery. Five patients had their operation performed open instead of laparoscopically for concerns surrounding aerosolized spread of SARS-CoV-2 during the initial days of the pandemic due to unavailability of air seals.

Eleven patients were offered adjuvant chemotherapy, but three refused in view of COVID-19 risks from immunosuppression and repeated hospital visits. Five patients completed adjuvant chemotherapy, of which four were alive at the end of two years despite two having upstaging in their second year and therapy for it. One of the three patients who refused adjuvant therapy died at the end of two years but the numbers were too small to calculate significance.

Two patients were offered palliative chemotherapy and one patient accepted the plan. However, both died at the end of the two years. Patients with delays in treatment were not found to be more likely to upstage within the first year or have a poorer outcome at the end of two years than those who had no delays (P=0.942), as shown in Figure-2. We attribute this to the fact that none were lost to follow-up and that the average duration of delay was less than five weeks, which in the larger scheme of things is small because the adenoma-carcinoma-spread sequence takes months to years. None of the covariates was found to be statistically significant in influencing delays or outcomes in the face of the pandemic. Five patients with malignant histology had COVID-19 during their hospitalization, of which one patient was positive on the swab performed on the day of discharge from the green non-NHS site (one out of 29), while four of the 12 patients managed at the NHS site had COVID-19 during admission (P<0.001).

No 30-day or operative mortalities were observed in this cohort. Eight patients died during the subsequent two years, four of whom had been upstaged during the two years despite adequate treatment; this was considered in line with the usual data for the UK population. Two patients managed at the green site required transfer to the NHS hospital because of the urgent need for intensive treatment unit (ITU) care, a facility that was not available at the private non-NHS site. All patients underwent a detailed preoperative discussion with their colorectal surgeons and informed consent was obtained prior to surgery and postoperative care at a non-NHS site. There was direct consultant supervision for all operated cases, including consultant-led daily rounds at the non-NHS site, which was treated as a nontraining location. Senior anesthesiologists and theatre staff had considerable prior NHS experience. There was a separate registrar rota set up for theatre assistance and continuous 24-hour in-hospital coverage by surgical registrars.

Six of the 41 patients had postoperative complications within the 30-day period, which were rated as Clavien-Dindo Grade II. None of the patients in the cohort had Grade III complications, while two patients required ITU care by default, classifying them as Grade IV but did not require interventional procedures. Two patients had a pulmonary embolism in the 30-day postoperative period despite prophylactic anticoagulation, and incidentally, both patients had COVID-19 during their hospitalization. Two consultants independently reviewed the entire management pathway for the cohort and deemed that eight of the 41 patients had a major change in the surgical plan due to the pandemic. Twenty-nine patients underwent surgery at a different site outside the NHS, which could also be considered a significant deviation from the usual. Multivariable logistic regression was used to investigate the likely relationship between treatment delay and the chance of a poor outcome. This occurred over the following two years and was as expected based on histologic TNM staging. The covariates included in the logistic model were age, sex, smoking status, BMI, TNM stage, and ASA status. The binary logistic regression model showed that COVID-related delays significantly affected treatment delays (P=0.015), but did not significantly affect outcomes. None of the other influential factors led to worse outcomes two years after the completion of treatment or the two-year mortality (Table-2).

## Discussion

This study assessed the effect of treatment delays and forced deviations from the usual pathway followed in the management of patients with proven and suspected colon cancer at a district general hospital in the UK during the first wave of COVID-19 pandemic. The NHS had released in 2020 for cancer management in face of the pandemic. These stated that a CRC treatment delay of up to 12 weeks is unlikely to have an impact on the outcomes [11].

Numerous studies have reported a significant reduction in new CRC cases during the early pandemic [12]. National lockdown, constraints in resources, and changing evidence showed the unacceptably high detrimental effect of perioperative COVID-19. This resulted in dramatic changes in oncologic management. Nearly all British hospitals experienced a sharp fall in the patient attendance at emergency rooms, primary care and specialist care with warning symptoms related to CRC during the lockdowns and an increase in the number of advanced visits related to CRC afterwards [13]. The number of MDT referrals was reduced compared to same five-month time frame from March through July 2019 in which we had received 52 new colon-cancer referrals. We received 55 and 66 referrals in 2022 and 2023, respectively.

A separate team and rota of NHS staff comprising consultants, registrars, surgical nurses, physiotherapists, and stoma nursers were established at the green site to maintain the standards of NHS cancer care. Nursing care, nutrition management, and theatre staff were non-NHS in many cases. The pandemic has mandated a change in the work of MDT meetings. Historically, most MDT meetings involved many participants gathering in the same room. However, the need for social distance and the effect of national governing bodies advocating working at home wherever possible have meant that virtual MDTs have become common practice, with all their benefits and drawbacks [14].

The results of an international survey also showed that treatment was delayed in more than 70% of CRC cases during the pandemic [15]. Urgent primary care referrals for suspected malignancy dropped by more than half in April 2020 compared with previous years, and by corollary, the estimated diagnostic delays may have led to an increase of more than 15% in avoidable deaths from CRC [13]. A study conducted during the first peak of COVID-19 in the UK showed only six out of 123 hospitals (5%) reported that their number of patient visits was still more than 90% of the usual number. In another study, the delay in diagnosis increased from 97 (12%) in 2019 to 136 (26%) in 2020.

As a result, the early diagnosis of cancer decreased significantly by more than 8%. Treatment of 23.4% of CRC patients was affected.13 In the UK, nearly 80%% of colorectal surgeons delayed their surgeries, and more than 10% stopped their surgeries. Sixty-nine percent performed their surgery in green sites which did not admit patients with symptoms related to the pandemic, 19.5% transferred their surgery to a a separate place, and only 11.5% continued to perform their surgery as usual. There has been a rapid adaptation to the multi-modality management of rectal cancer in the UK in response to the pandemic [16]. This has been well presented in the IMPACT-ReCaP study and the National Bowel Cancer Audit COVID-19 statement [10]. Rectal cancer patients from our centre were a part of this national audit as well.

Several studies have failed to demonstrate that treatment delays influenced disease progression and patient survival. Reasons suggested for this were related to the pathophysiology of colon cancer. This includes the time period between progression from adenomas to cancer, which takes between five and 15 years [16]. As such, a one- to three-month delay makes little difference in overall outcome. In addition, differences in the biology of cancers (i.e., some cancers are growing faster) may be more responsible for upstaging of patients rather than a delay in investigations or treatment.

However, we did not find any studies correlating deviations in the pathway with longer-term outcomes such as two years’ survival, except one from a neighboring NHS trust, which also assessed similar outcomes but with different parameters and from a standpoint of tumour staging [17].

Another NHS trust in the UK has assessed the impact of delays due to the pandemic on colon cancer treatments and on upstaging and psychological effects on patients. It was found that 46.7% of patients received treatment within 62 days of treatment. There was a statistically significant upstaging of the tumor in patients with delayed treatment [17]. However, there was no difference in the level of anxiety between the two groups of patients. Analysis of our database also showed that despite well-documented delays and changes to the usual colon cancer pathway, logistic regression failed to show any negative effect of these delays on outcomes two years after the end of the first lockdown and after completing the initial management phase of these patients.

Our study has several strong points. We prospectively maintained clinical records which were used to compare data, resulting in real-world evidence on the impact of the pandemic and deviations from standard care on colon cancer outcomes. The database was prospectively maintained in an MDT format and had direct entries available from the Somerset Cancer Registry database for correlation with outcomes. The completeness of the patient records allowed us to account for confounding factors which are the bane of such studies. However, one of the limitations of our study was its small sample size.

Our sample size was limited by adhering to the number of colon cancer patients admitted through the two-week wait-referral system in a moderate-sized general hospital during the pandemic. It was also limited by the reduced number of referrals due to strain on general practitioners and reluctance of patients to present to hospitals or primary care for non-life-threatening symptoms. Another possible limitation is the unavailability of an on-site intensive care unit or blood bank. All patients were informed of these limitations and ways to overcome them by accepting the risk of urgent transfer to the NHS site less than half a mile away, in case it was required. There was a clear change in the consenting process to reflect the above details with the option of surgery at the existing NHS site if the patient was willing to accept the risk of being in the same bay or ward as COVID positive patients.

## Conclusion

A subset of patients had changes to their surgical plans in addition to other alterations or delays in their two-week wait and 62-day target pathways during the COVID-19 pandemic. We assessed the effects of all these changes and delays and found significant delays in their immediate management but no significant negative effect on their two-year outcomes.

Eight percent of cancer surgeries performed during the first lockdown could have been avoided if endoscopic visualization and biopsy were performed along the usual pathway, and 8% of surgeries performed open could have been performed laparoscopically without fear of surrounding aerosol-generating procedures at the start of the pandemic. Significant changes were made to the usual management pathway for colon cancer patients at our district general NHS hospital in an attempt to maintain the targets set for these patients within the realms of the lower gastrointestinal MDT during the period of lockdown initiated during the first wave. Lessons learned from such data can be used to advise on the urgent changes required in the case of future emergencies.

## Acknowledgement

We acknowledge the work of the MDT and hospital staff at the NHS site and at the private site for patient care and their accurate record-keeping during the testing times of the early pandemic and the following two years.

## Conflict of Interest

None.
